# Supplementary material for: White Tea Reduces Dyslipidemia, Inflammation, and Oxidative Stress in the Aortic Arch in a Model of Atherosclerosis Induced by Atherogenic Diet in ApoE Knockout Mice
Source: Pharmaceuticals (Basel). 2024 Dec 17;17(12):1699. doi: 10.3390/ph17121699 (PMC11679696; doi:10.3390/ph17121699)
Supplement: Supplementary file 1 [file pharmaceuticals-17-01699-s001.zip › pharmaceuticals-3337353-supplementary.pdf]

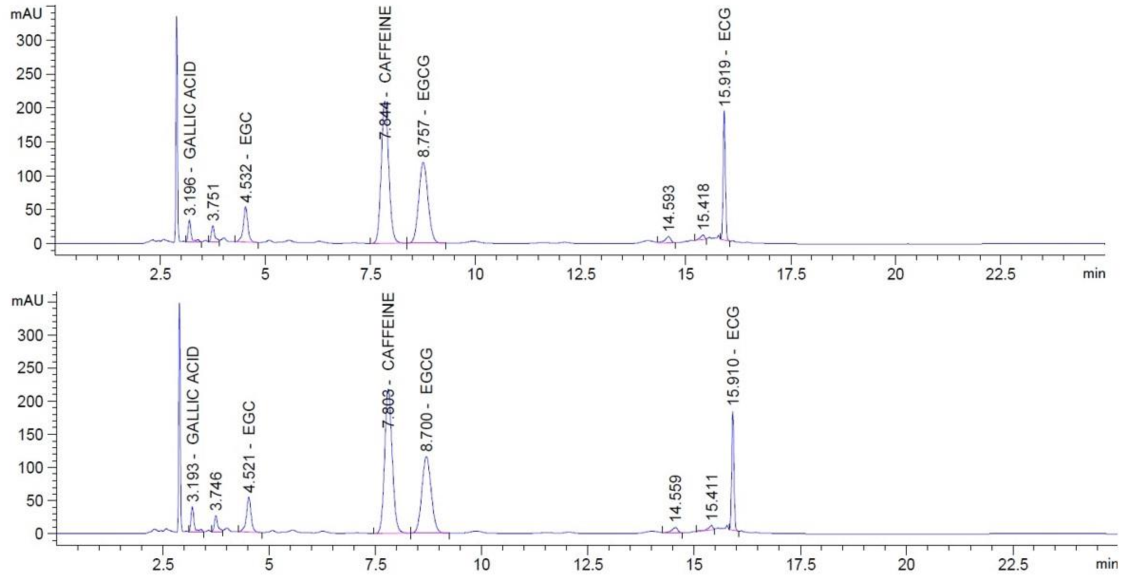

Figure S1. HPLC-DAD chromatogram of standarts.

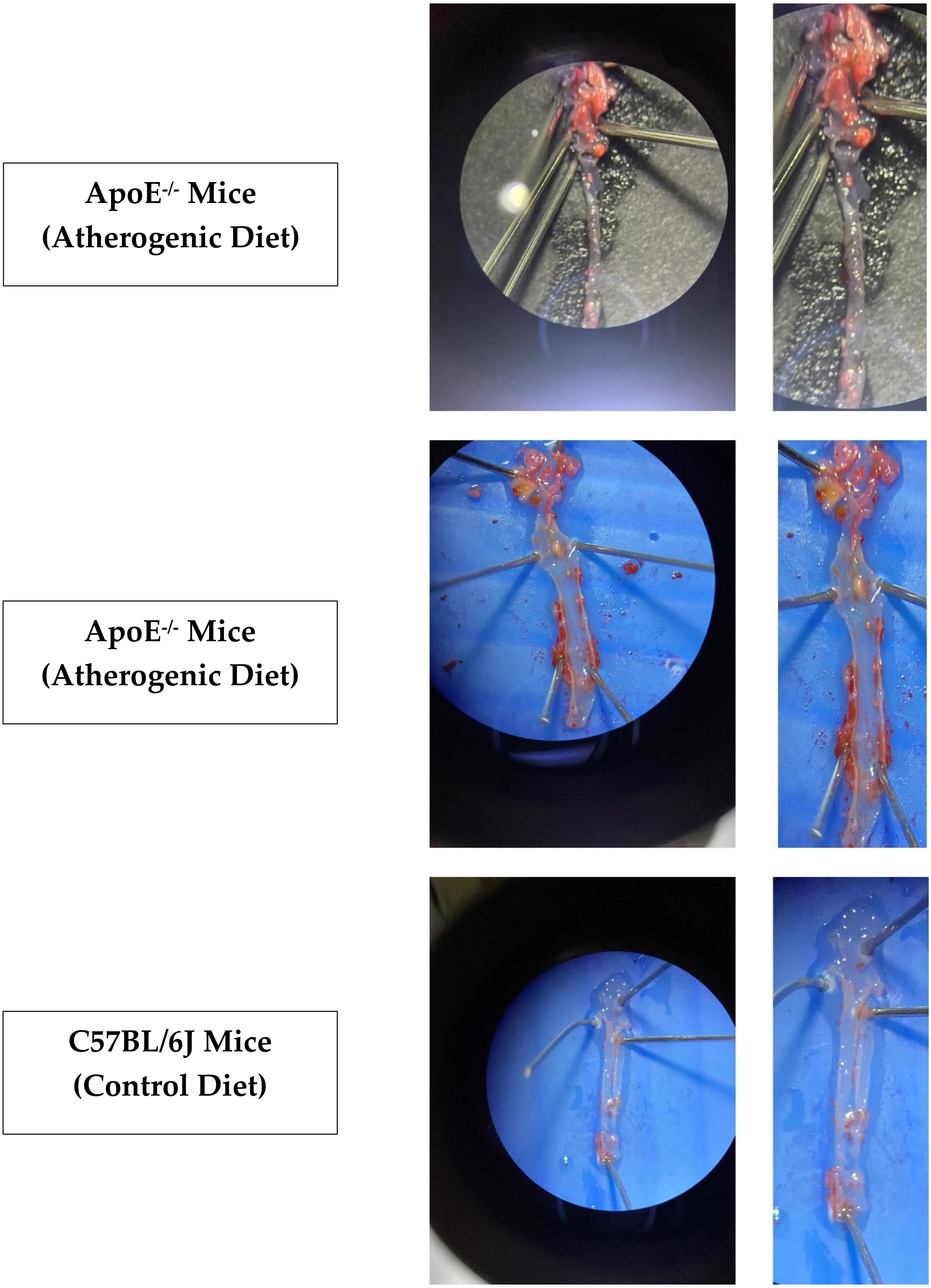

Figure S2. Aortic Tissue Oil Red O (ORO) en face staining results.
